# Redescription of holotypes of four *Alopecosa* species (Araneae, Lycosidae) from China

**DOI:** 10.3897/zookeys.945.52287

**Published:** 2020-07-03

**Authors:** Jia Tang, Xiang Xu, Haiqiang Yin, Yuri M. Marusik, Zongguang Huang

**Affiliations:** 1 College of Life Science, Hunan Normal University, Changsha 410081, Hunan, China Hunan Normal University Changsha China; 2 Institute for Biological Problems of the North RAS, Portovaya Str. 18, Magadan 685000, Russia Institute for Biological Problems of the North RAS Magadan Russia; 3 Department of Zoology and Entomology, University of the Free State, Bloemfontein 9300, South Africa University of the Free State Bloemfontein South Africa; 4 Zoological Museum, Biodiversity Unit, University of Turku, FI-20014, Finland University of Turku Turku Finland

**Keywords:** Lycosinae, new synonymy, redescription, wolf spiders

## Abstract

The holotypes of four species of *Alopecosa* Sundevall, 1833 described from China, *A. disca* Tang, Yin & Yang, 1997 (♀); *A. orbisaca* Peng, Yin, Zhang & Kim, 1997 (♀); *A. wenxianensis* Tang, Yin & Yang, 1997 (♂), and *A. xilinensis* Peng, Yin, Zhang & Kim, 1997 (♀), are reexamined. Detailed descriptions, illustrations, remarks, and a distribution map of the three valid species are given. *Alopecosa xilinensis***syn. nov.** is found to be junior synonym of *Alopecosa licenti* (Schenkel, 1953).

## Introduction

*Alopecosa* Simon, 1885, with 162 valid named species (WSC 2020), is the third largest genus in Lycosidae. Only *Pardosa* C.L. Koch, 1847 (542 species) and *Arctosa* C.L. Koch, 1847 (171) have more species (WSC 2020). *Allopecosa* is considered as globally distributed genus, known from all biogeographical realms, but most of its species occur in the Palaearctic. The genus is relatively poorly studied: over at third of all species (67) are known by a single sex, and *Alopecosa* has never been revised at a wide scale. There are only regional reviews of the genus in Europe (Lugetti and Tongiorgi 1969), the Nearctic region (Dondale and Redner 1979), and Japan (Tanaka 1992). The lack of the studies on *Alopecosa* is partly caused by the brief original descriptions, some of which lack figures, and by difficult access to type material in numerous museums. Currently, 42 *Alopecosa* species are known in China (WSC 2020). Of them, 17 are known by a single sex and 12 are known by a single taxonomic entry (WSC 2020). Leading up to a regional revision of *Alopecosa* from East Asia, we redescribe all available types of poorly known species deposited in Chinese institutions. The goal of this paper is to provide detailed illustrated redescription of four species deposited in the Hunan Normal University.

## Material and methods

Specimens were examined under an Olympus SZX16 stereomicroscope and an Olympus BX53 compound microscope. Photographs were taken with a Canon PowerShot G12 digital camera mounted on an Olympus BX53 compound microscope. Both the male palps and female genitalia were examined, photographed, and illustrated after being dissected. All morphological measurements are calculated using a stereomicroscope (LEICA M205C) and given in millimeters. Eye diameters are taken at the widest point. Promarginal and retromarginal teeth on the chelicerae are given as the first, second, third, etc., from the base of the fang to the distal groove.. Leg measurements are given as total length (femur, patella, tibia, metatarsus, tarsus). Measurements of the holotypes are from the original description. All specimens examined in this study are deposited in the College of Life Sciences, Hunan Normal University (HNU).

Terminology in the present paper follows Zyuzin (1993) and Nadolny (2018). The abbreviations used in the present paper are as follows:

**Ag** accessorial gland;

**ALE** anterior lateral eye;

**AME** anterior median eye;

**AME–AME** distance between AMEs;

**AME–ALE** distance between AME and ALE;

**At** atrium;

**Cd** copulatory duct;

**Em** embolus;

**Et** tip of embolus;

**Fd** fertilization duct;

**Ho** hood;

**Ma** median apophysis;

**Pa** palea;

**PLE** posterior lateral eye;

**PME** posterior median eye;

**PME–PLE** distance between PME and PLE;

**PME–PME** distance between PMEs;

**Sb** septal base;

**Sd** sperm duct;

**Sp** spermatheca;

**Ss** septal stem;

**STL** sternum length;

**STW** sternum width;

**Sy** synembolus;

**Ta** tegular apophysis.

## Taxonomy

**Family Lycosidae Sundevall, 1833**

### Genus Alopecosa

Taxon classificationAnimaliaAraneaeLycosidae

Simon, 1885

FD84B1E0-CBD4-5BEF-87D6-601AD9F22D34

#### Type species.

*Araneus fabrilis* Clerck, 1757 from Sweden.

### Alopecosa disca

Taxon classificationAnimaliaAraneaeLycosidae

Tang, Yin & Yang, 1997

0D1636B7-6B99-5904-A14B-2684276A5ADB

Figures 1 , 2 , 10

Alopecosa disca Tang, Yin & Yang in Yin et al. 1997: 64, fig. 27a–d (♀); Song et al. 1999: 317, fig. 187E (♀, republication of the original figures).

#### Type.

***Holotype*** ♀ (HNU, Lyco-*Alop*-0004-001): China, Gansu Province, Lanzhou City, Yuzhong County, Temple Majiamiao, 13.VII.1981, leg. Yingqiu Tang. Temple Majiamiao: 35°51'N, 104°7.12'E (information supplied by present authors).

#### Diagnosis.

**Figure 1.**
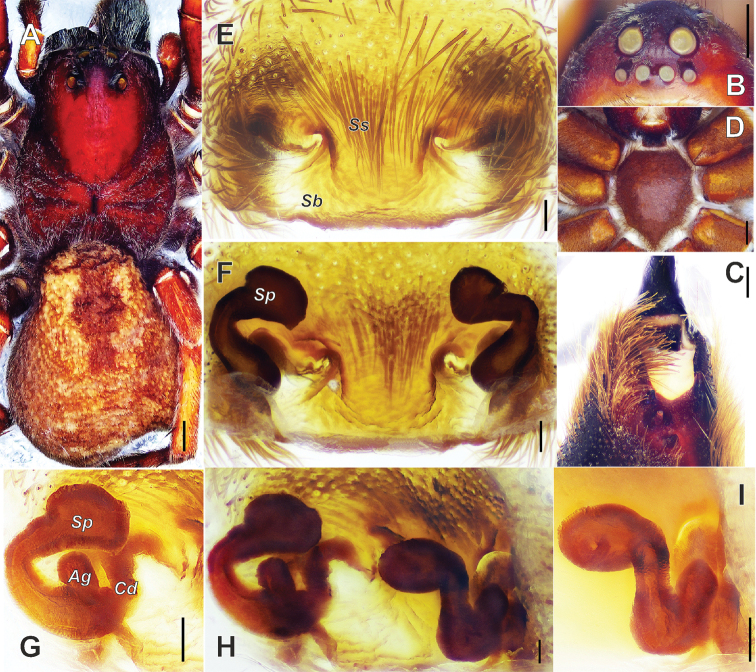
*Alopecosa disca* Peng, Yin, Zhang & Kim, 1997, female. **A** Habitus, dorsal view **B** eyes, front view **C** chelicera, ventral view **D** sternum, ventral view **E** epigyne **F** vulva **G** vulva, showing the detailed left half **H** vulva, moved slightly from the normal ventral view **I** vulva, showing the detailed right half. Abbreviations: ***Ag*** accessorial gland, ***Cd*** copulatory duct, ***Sb*** septal base, ***Sp*** spermatheca, ***Ss*** septal stalk. Scale bars: 1 mm (**A, B, D**); 0.5 mm (**C**); 0.1 mm (**E–I**).

Epigyne of this species is similar to that of *A. chagyabensis* Hu & Li, 1987, a species known from Xizang Autonomous Region of China. Both species are lacking anterior hood, have base of septum width much longer than septal stem length, both have distinct copulatory opening, but can be separated by septum width/length ratio 2.5 in *A. disca* and 1.8 in *A. chagyabensis*. Two species well differ by the shape of the endogyne (compare Fig. 1F–I and fig. 64-2 in Hu 2001).

#### Description.

Body (Fig. 1A) length 16.0, carapace 7.9 long, 5.0 wide, abdomen 8.8 long, 5.8 wide (data from original description by Yin et al. 1997: 64). Carapace dark reddish brown. Cervical and radial grooves distinct, darker than body color. Fovea linear, short, but a little thick. Head region wide, with anterior margin almost 4/5 width of thorax region. Anterior eye row straight, almost as wide as median one, posterior row widest. Eye sizes and inter-distances (Fig. 1A, B): AME 0.30, ALE 0.27, PME 0.49, PLE 0.47; AME–AME 0.19, AME–ALE 0.2, PME–PME 0.49, PME–PLE 0.67. Clypeus height 0.23. Chelicerae black-brown, with three promarginal and two retromarginal teeth (Fig. 1C). Both labium and endites black brown, with reddish brown distal parts. Sternum (Fig. 1D) brown, with metallic luster. STL 3.52, STW 2.82 (Fig. 1D). Palp and legs reddish brown, robust, without any distinct annuli. Leg measurements: I 18.30 (5.00, 6.60, 3.80, 2.90); II 16.70 (4.80, 5.80, 3.60, 2.50); III 15.00 (4.50, 5.00, 3.60, 1.90); IV 20.40 (6.10, 7.00, 5.00, 2.30) (data from original description by Yin et al. 1997: 64), leg formula 4123. Dorsum of abdomen (Fig. 1A) dark brown mixed yellowish brown. Cardiac mark distinct, dark brown and large. Posterior half of abdomen with 4 or 5 chevrons (because the type specimen has been wrinkled, chevron patterns showed in color photos of the present paper are not clearer than those showed in line drawings of the original paper). Venter of abdomen grey yellowish in the middle and dark grey laterally.

**Figure 2.**
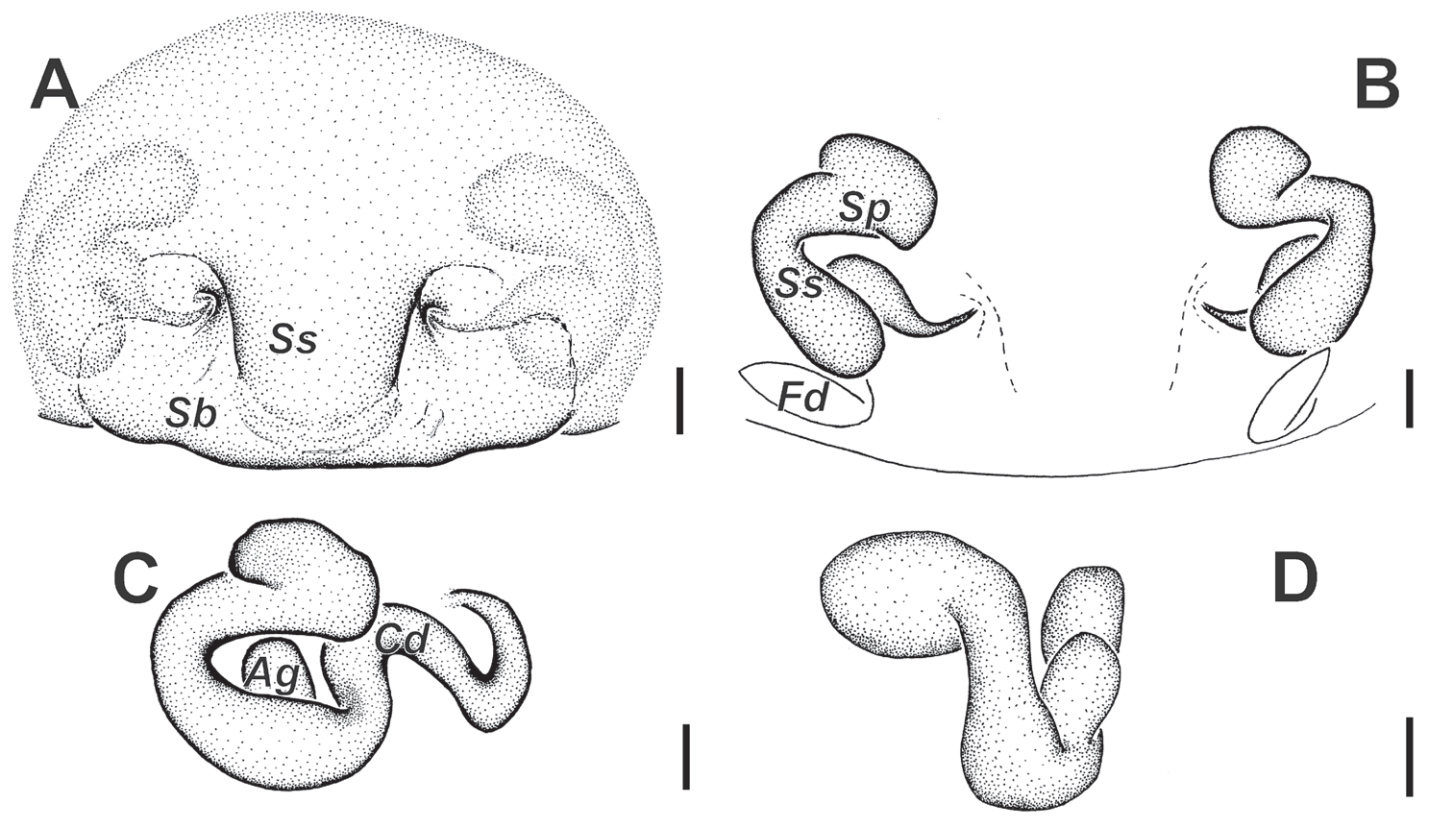
*Alopecosa disca* Peng, Yin, Zhang & Kim, 1997, female. **A** Epigyne **B** vulva **C** vulva, showing the detailed left half **D** vulva, showing the detailed right half. Abbreviations: ***Ag*** accessorial gland, ***Cd*** copulatory duct, ***Fd*** fertilization duct, ***Sb*** septal base, ***Sp*** spermatheca, ***Ss*** septal stalk. Scale bars: 0.1 mm (**A–D**).

Epigyne (Figs 1E–I, 2) wider than long. Atrium and anterior hood absent. Septum weakly sclerotized, with short stem (*Ss*) and broad base (*Sb*); stem shorter than base height. Spermathecae with clavate head, slender and curved stalk, and accessorial gland (*Ag*) situated at the place near copulatory duct. Copulatory ducts short, slightly twisted.

**Male.** Unknown.

#### Distribution.

Only known from the type locality, Gansu, China (Fig. 10).

### Alopecosa orbisaca

Taxon classificationAnimaliaAraneaeLycosidae

Peng, Yin, Zhang & Kim, 1997

E202859B-67ED-54E6-B587-8FA054319586

Figures 3 , 4 , 10

Alopecosa orbisaca Peng et al. 1997: 41, figs 1–5 (♀); Yin et al. 1997: 70, fig. 30a–e (♀, republication of the original figures); Song et al., 1999: 317, fig. 187M (♀, republication of the original figure).

#### Type.

***Holotype*** ♀ (HNU, Lyco-*Alop*-0003-001): China, Qinghai Province, Xining City, 36°36'N, 101°48'E, 1978.

#### Diagnosis.

**Figure 3.**
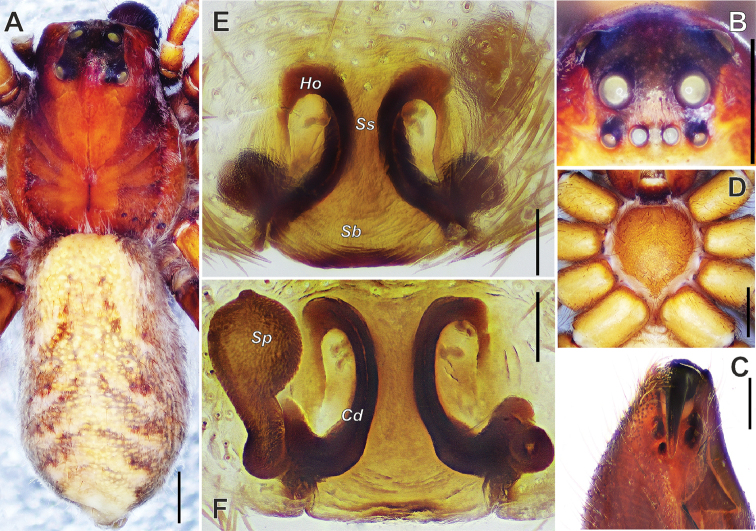
*Alopecosa orbisaca* Peng, Yin, Zhang & Kim, 1997, female. **A** Habitus, dorsal view **B** eyes, front view **C** chelicera, ventral view **D** sternum, ventral view **E** epigyne **F** vulva (right spermatheca lost). Abbreviations: ***Cd*** copulatory duct, ***Ho*** hood, ***Sb*** septal base, ***Sp***-spermatheca, ***Ss*** septal stalk. Scale bars: 1 mm (**A, B, D**); 0.5 mm (**C, E, F**).

The female of this species is similar to that of *Alopecosa zyuzini* Logunov & Marusik, 1995 in having a pair of separate anterior hoods and similar shape of septum. *Alopecosa orbisaca* can be separated from similar species by having large size (carapace 4.0 vs 2.85–3.58 in *A. zyuzini*), and wider stem of septum (septum wider than hood vs narrower than hood) (compare Fig. 3E and fig. 64-2 in Hu 2001).

#### Description.

***Holotype*** female. Body (Fig. 3A) length 9.20, carapace 4.00 long, 3.50 wide, abdomen 5.2 long, 3.20 wide (data from original description in Peng et al., 1997: 41). Carapace brown, with median pattern orange brown, short and about 1/4 width of thorax region. Cervical and radial grooves black brown, distinct. Pair of paraxial longitudinal bands brown, each band about 1/4 width of thorax region. Pair of lateral longitudinal bands also brown, however each band half of width of paraxial longitudinal band. Fovea long, linear, black. Cephalic region wide, with anterior margin about 3/4 width of thorax region. Anterior eye row straight, as wide as median one, posterior row widest. Eye sizes and interdistances (Fig. 3A, B): AME 0.14, ALE 0.16, PME 0.3, PLE 0.18; AME-AME 0.13, AME-ALE 0.11, PME-PME 0.35, PME-PLE 0.51. Clypeus height 0.19. Chelicerae black, with 3 promarginal (the first one largest and the third one smallest) and 2 retromarginal teeth (Fig. 3C). Labium with the base black and the distal part yellow and endites black-brown. Sternum yellow-brown, STL 1.94, STW 1.81 (Fig. 3D). Palps and legs reddish brown, robust, no distinct annulus except for femur of palp with dark stripes dorsally. Leg measurements: I 9.80 (2.90, 3.20, 2.00, 1.70); II 8.70 (2.80, 2.70, 1.90, 1.30); III 8.70 (2.80, 2.70, 1.90, 1.30); IV 11.30 (3.10, 3.50, 2.80, 1.90) (data from original description by Peng et al. 1997: 41). Abdomen yellow-brown, with large, pale cardiac and 5 brown chevron patterns dorsally (Fig. 3A). Venter of abdomen grey brown.

**Figure 4.**
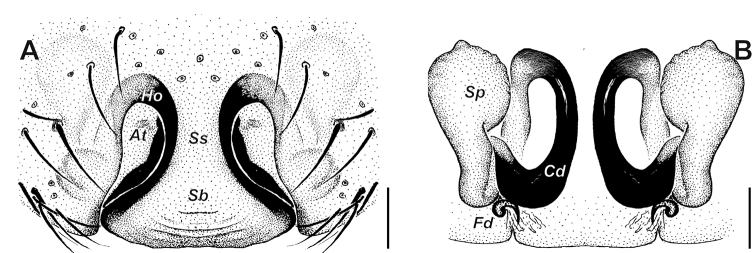
*Alopecosa orbisaca* Peng, Yin, Zhang & Kim, 1997, female. **A** Epigyne **B** vulva. Abbreviations: ***At*** atrium, ***Cd*** copulatory duct, ***Fd*** fertilization duct, ***Ho*** hood, ***Sb*** septal base, ***Sp*** spermatheca, ***Ss*** septal stalk. Scale bars: 0.1 mm.

Epigyne (Figs 3E, F, 4) wide than long, with septum as long as wide, stem gradually widening toward the base, lateral margins of septum strongly sclerotized; anterior part of atrium with a pair of hoods (*Ho*); anterior part of septum (or stem, *Ss*) about 1.5 times wider than hoods. Spermatheca (*Sp*) divided distinctly into head and stalk, both of them with not smooth surface; a strong twist present at the connecting part between spermatheca and copulatory duct (*Cd*); copulatory duct long, ear-shaped.

**Male.** Unknown.

#### Distribution.

Only known from the type locality, Qinghai, China (Fig. 10).

### Alopecosa wenxianensis

Taxon classificationAnimaliaAraneaeLycosidae

Tang, Yin & Yang, 1997

601A5603-FFC8-545D-9360-AC40F750D6E9

**Figure 5.**
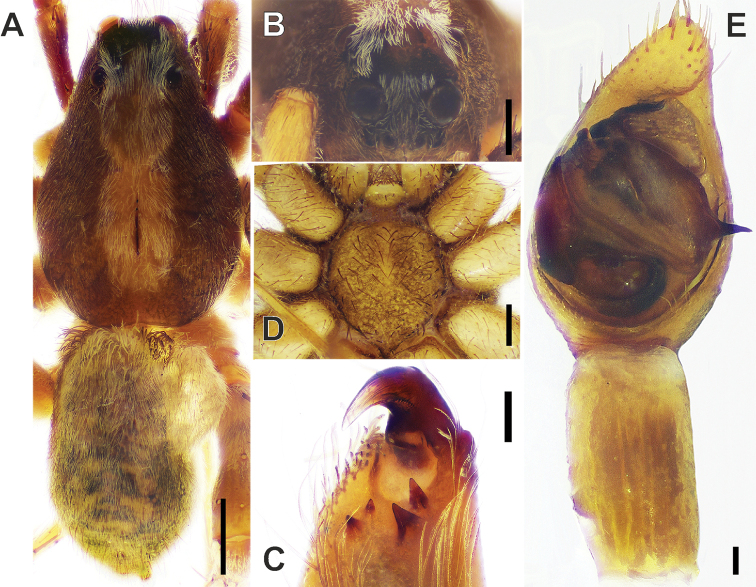
*Alopecosa wenxianensis* Tang, Yin & Yang, 1997, male. **A** Habitus, dorsal view **B** eyes, front view **C** chelicera, ventral view **D** sternum, ventral view **E** palp, ventral view (showing complete tibia). Scale bars: 1 mm (**A**); 0.5 mm (**B, D**); 0.1 mm (**C, H**).

**Figure 6.**
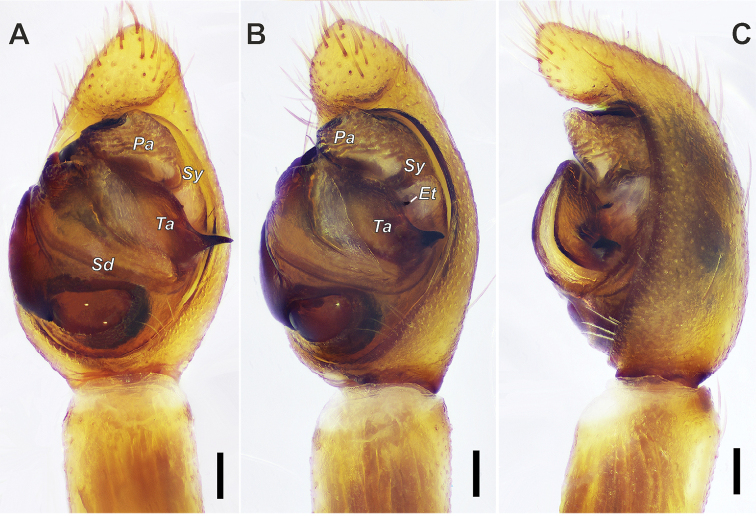
*Alopecosa wenxianensis* Tang, Yin & Yang, 1997, male. **A** Palp, ventral view **B** palp, ventral-retrolateral view **C** palp, retrolateral view. Abbreviations: ***Et*** tip of embolus, ***Pa*** palea, ***Sd*** sperm duct, ***Sy*** synembolus, ***Ta*** tegular apophysis. Scale bars: 0.1 mm.

**Figure 7.**
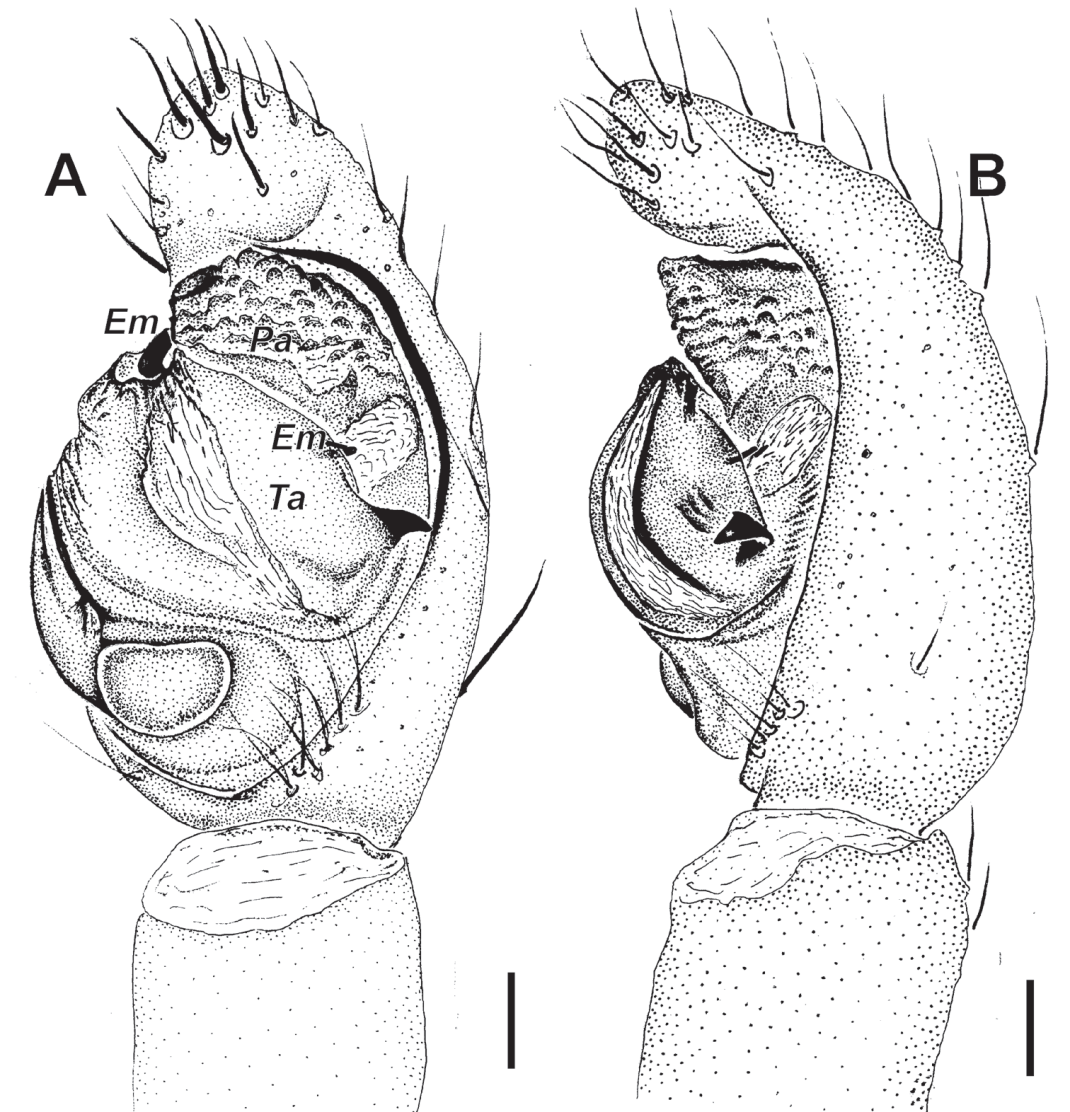
*Alopecosa wenxianensis* Tang, Yin & Yang, 1997, male. **A** palp, ventral view **B** same, retrolateral view. Abbreviations: Em-embolus; ***Ta***-tegular apophysis; ***Pa***-palea. Scale bars: 0.1 mm.

Figures 5 , 6 , 7 , 10

Alopecosa wenxianensis Tang, Yin & Yang in Yin et al. 1997: 75, fig. 33a–e (♂); Tang et al. 1998: 91, fig. 2a–e (♂); Song et al. 1999: 318, fig. 188J (♂, republication of the original figure).

#### Type.

***Holotype*** ♂ (HNU, Lyco-*Alop*-0001-001): China, Gansu Province, Longnan City, Wenxian County (32°54'N, 104°6'E), Guanjiagou, 2.V.1992, leg. Yingqiu Tang.

#### Diagnosis.

Male palp of this species (Figs 5E, 6, 7) is similar to that of *A. xinjiangensis* Hu & Wu, 1989 by having sharply pointed tegular apophysis and differs by having relatively shorter tip of cymbium, rounded anterior edge of the tegular apophysis (vs almost straight) and tip of tegular apophysis located in mid part of the bulb (vs anterior 1/3) (Hu and Wu 1989: figs 162.5–6; Marusik et al. 2007: fig. 3).

#### Description.

Body (Fig. 5A) length 7.9, carapace 4.4 long, 3.0 wide, abdomen 3.5 long, 2.2 wide (after Yin et al. 1997: 75). Carapace dark-brown, with median pattern yellow-brown and about 1/3 width of thorax region. Cervical groove and radial grooves distinct, slightly darker than body color. A pair of lateral longitudinal bands dark brown, each about 2/3 width of thorax region. Cephalic region with anterior margin about 1/3 width of thoracic part. Anterior eye row slightly recurved, slightly narrower than median one, posterior row widest. Ocular area covered with white setae; eye sizes and inter-distances (Fig. 5A, B): AME 0.12, ALE0.13, PME 0.31, PLE 0.25; AME-AME 0.12, AME-ALE 0.1, PME- PME 0.31, PME-PLE 0.38. Clypeus height 0.09. Chelicerae brown, with blackish-grey patterns, and with 2 small promarginal and 2 large retromarginal teeth (Fig. 5C). Labium and endites yellow-brown. Sternum brown, with marginal lines darker, STL 1.3, STW 1.04 (Fig. 5D). Palp and legs brown, with black and gray patterns. Leg measurements: I 10.2 (2.8, 3.4, 2.5, 1.5); II 9. 80 (2.6, 3.4, 2.3, 1. 5); III 10. 0 (2.5, 3.3, 2.6, 1.6); IV 13.5 (3.7, 4.2, 3.8, 1.8) (after Yin et al., 1997: 75). Dorsum of abdomen dark brown, densely covered with setae. Cardiac mark about 1/3 abdomen length, black brown, with several yellow-brown transversal or oblique stripes on the sides of it. Venter of abdomen yellow-brown medially, and with irregular grey-black dots laterally.

Palp (Figs 5E, 6, 7). Cymbium brown, about 1.5 times longer than tibia, ca 1.8 times longer than wide, with the tip slightly swollen and covered with some strong setae. Bulb 1.25 times longer than wide; tegulum inclined at ca 50º angle and sperm duct (*Sd*) at about 20°; tegular apophysis sharply pointed, with smoothly rounded anterior margin, tip directed at right angle to the axis of cymbium and located in the middle part of the bulb; palea subequal in size to the subtegulum, with almost undeveloped short and rounded synembolus (*Sy*); embolus hidden by the tegular apophysis and only tip visible in ventral view.

**Female.** Unknown.

#### Distribution.

Known only from the type locality, Gansu, China (Fig. 10).

#### Remarks.

Because the abdomen is wrinkled, several transversal or oblique stripes on the dorsum shown in Figure 5A are less clear than those shown in line drawings of the original description.

### Alopecosa licenti

Taxon classificationAnimaliaAraneaeLycosidae

(Schenkel, 1953)

E439D523-0408-5FDD-9AF6-5B670387119E

Figures 8 , 9

Tarentula licenti Schenkel 1953: 77, fig. 36 (♀).
Tarentula argentata Schenkel 1963: 306, fig. 174a, b (♂).
Tarentula fenestrata Schenkel 1963: 311, fig. 177 (♀).
Tarentula fenestrata pseudobarbipes Schenkel 1963: 312, fig. 178 (♀).
Tarentula davidi Schenkel 1963: 313, fig. 179 (♀).
Tarentula orbiculata Schenkel 1963: 315, fig. 180 (♀).
Tarentula bipennis Schenkel 1963: 316, fig. 181 (♀).
Alopecosa xilinensis Peng, Yin, Zhang & Kim 1997: 42, figs 6–9 (♀); Yin et al. 1997: 76, fig. 34a–d (♀, republication of figures from Peng et al. 1997); Song et al. 1999: 318, fig. 188D (♀, copy of fig. 30c in Yin et al. 1997), syn. nov. For complete list of references, see WSC (2020).

#### Material examined.

***Holotype*** ♀ of *Alopecosa xilinensis*Peng et al., 1997 (HNU, Lyco-*Alop*-0002-001) from China, Inner Mongolia Autonomous Region, Xilinhot, 44°N, 116°6'E, 13–15.VII.1987, leg. Jiafu Wang; 3 ♀, Inner Mongolia, VII.1983, leg. Lita Wu.

#### Remarks.

**Figure 8.**
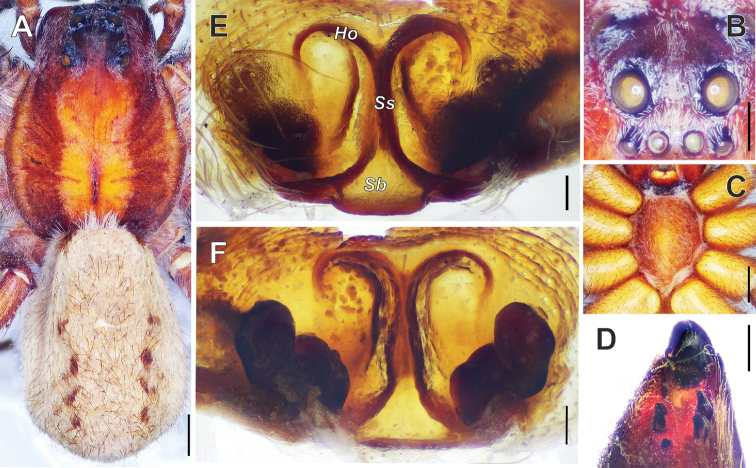
*Alopecosa licenti* (Schenkel, 1953), female. **A** Habitus, dorsal view **B** eyes, front view **C** Sternum, ventral view **D** Chelicera, ventral view **E** Epigyne **F** Vulva. Abbreviations: ***Ho***-hood; ***Sb***-septal base; ***Ss***-septal stem. Scale bars: 1 mm (**A–C**); 0.5 mm (**D**); 0.1 mm (**E, F**).

**Figure 9.**
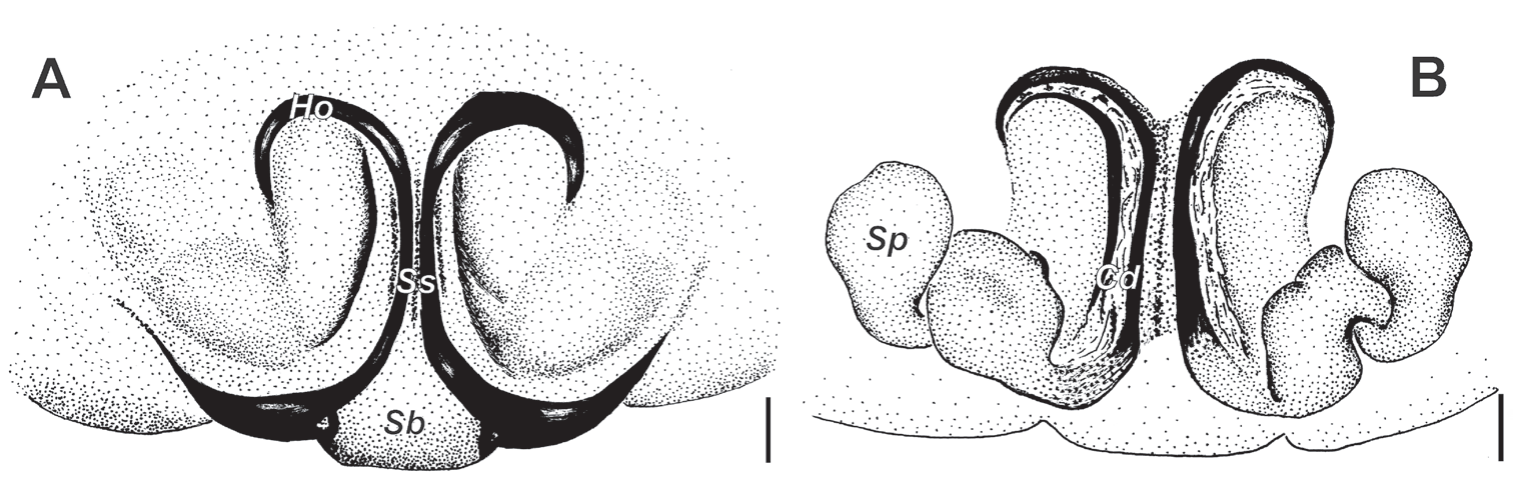
*Alopecosa licenti* (Schenkel, 1953), female. **A** Epigyne **B** vulva. Abbreviations: ***Cd***-copulatory duct; ***Ho***-hood; ***Sb***-septal base; ***Sp***-spermathecae; ***Ss***-septal stem. Scale bars: 0.1 mm.

Copulatory ducts in the original description of the species are known short and stout (Peng et al. 1997: 42). In fact, they are long and slender, and the short and stout parts are stalks of the spermathecae (Figs 8F, 9B).

#### Comments.

This species has the most synonyms of any Chinese *Alopecosa* due to variations of the shape of the epigyne. Some individuals have very narrow septum (Schenkel 1953: fig. 36; Schenkel 1963: figs 177, 179; Yin et al. 1997: fig. 29b; Zhu and Zhang 2011: fig. 183A) and some have slightly wide septum (Schenkel 1963: figs 178, 180, 181; Song 1986: fig. 14). The septum has a smooth posterior margin (Schenkel 1953: fig. 36; Schenkel 1963: figs 179–181; Yin et al. 1997: fig. 29b; Zhu and Zhang 2011: fig. 183) in some specimens. But in others, the posterior margin of the septum has a lip-shaped protrusion (Schenkel 1963: figs 177–178). The holotype of *A. xilinensis* has the same narrow septum as shown by Schenkel (1963: fig. 177) or by Yin et al. (1997: fig. 29b). It also has the same posterior margin of septum as shown by Schenkel (1963: fig. 178). The vulva of holotype of *A. xilinensis* is the same as figured by Song (1986: fig. 15), Yin et al. (1997: fig. 29c), and Zhu and Zhang (2011: fig. 183b). Accounting for the similarity of the epigyne and vulva of the holotypes of *A. xilinensis* and *A. licenti*, we consider these names to be synonyms.

#### Distribution.

The species has a rather wide distribution in China, known from Gansu to Heilongjiang and south to Sichuan (Zhu and Zhang 2011; Li and Lin 2016). Besides China, this species is known from Tuva, Khabarovsk and Maritime provinces in Russia (Mikhailov 2013), Mongolia, and also Korea (WSC 2020).

**Figure 10.**
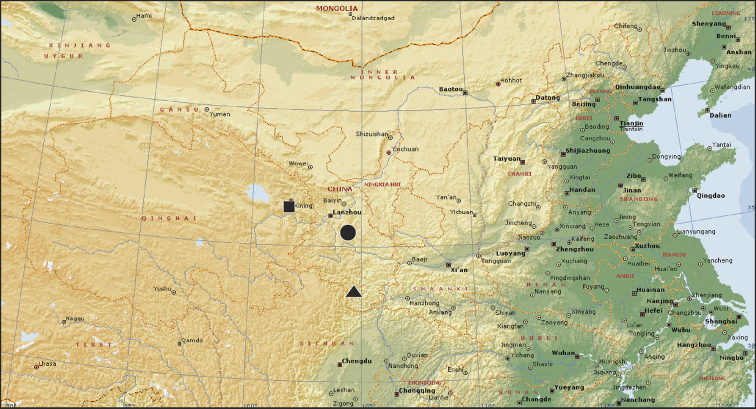
Type localities of *Alopecosa disca* (circle), *Alopecosa orbisaca* (square), and *Alopecosa wenxianensis* (triangle).

## Supplementary Material

XML Treatment for Alopecosa

XML Treatment for Alopecosa disca

XML Treatment for Alopecosa orbisaca

XML Treatment for Alopecosa wenxianensis

XML Treatment for Alopecosa licenti
